# Biliary Atresia Associated With Jejunal Atresia: A Case Report

**DOI:** 10.7759/cureus.65463

**Published:** 2024-07-26

**Authors:** Edmund Choong Yew Hoe, Mohd Shahrulsalam Mohd Shah, Ikhwan Sani Mohamad, Voon Meng Leow

**Affiliations:** 1 Surgery, Universiti Sains Malaysia, Kota Bharu, MYS; 2 Surgery, Hospital Universiti Sains Malaysia, Kota Bharu, MYS; 3 Paediatric Surgery, Universiti Sains Malaysia, Kota Bharu, MYS; 4 Paediatric Surgery, Hospital Universiti Sains Malaysia, Kota Bharu, MYS; 5 Surgery, Advanced Medical and Dental Institute, Universiti Sains Malaysia, Kepala Batas, MYS

**Keywords:** cholangiopancreatography, kasai procedure, hyperbilirubinaemia, biliary atresia, jejunal atresia

## Abstract

A girl who was born at 40 weeks of gestation weighing 3800 g presented with bilious vomiting and abdominal distension shortly after birth. A lower gastrointestinal contrast study showed a microcolon with small bowel atresia. Subsequently, laparotomy, small bowel resection and anastomosis were done. Intra-operative findings noted jejunal atresia type 3a. Post-operatively, the patient developed persistent conjugated hyperbilirubinaemia and hence, magnetic resonance cholangiopancreatography (MRCP) was performed. MRCP revealed possible biliary atresia (BA) of which the patient underwent Kasai hepato-porto-enterostomy. We reported a rare case of double pathology involving jejunal atresia and BA, describing its aetiology, characteristics and treatment availability based on literature.

## Introduction

Jejuno-ileal atresia (JIA) is a rare congenital anomaly causing the obstruction of small bowels in newborns. Incidence varies between 1:330 to 1:3000 live births and jejunal atresia accounts for 40-50% of the cases [1,2]. JIA is widely believed to stem from an intrauterine ischemic event impacting the midgut, resulting in the occlusion of one or more segments of the developing intestine. This ischemic insult can trigger necrosis within the affected bowel segments, ultimately leading to their resorption or narrowing. While the precise aetiology of this vascular disruption remains under investigation, it is thought to involve a complex interplay of genetic predisposition and environmental factors [3]. Although JIA is associated with numerous congenital anomalies, biliary atresia (BA) which is a progressive inflammatory and obstructive pathology affecting the bile ducts, is extremely rare. The inflammatory component of BA involves immune-mediated responses, although the exact aetiology remains incompletely understood. Genetic predispositions, viral infections, and autoimmune processes have been implicated in the pathogenesis of this condition, suggesting a multifactorial interplay of factors contributing to bile duct injury and fibrosis. We report a rare case of double pathology involving jejunal and BA, describing its aetiology, characteristics and treatment availability based on literature.

## Case presentation

**Figure 1:**
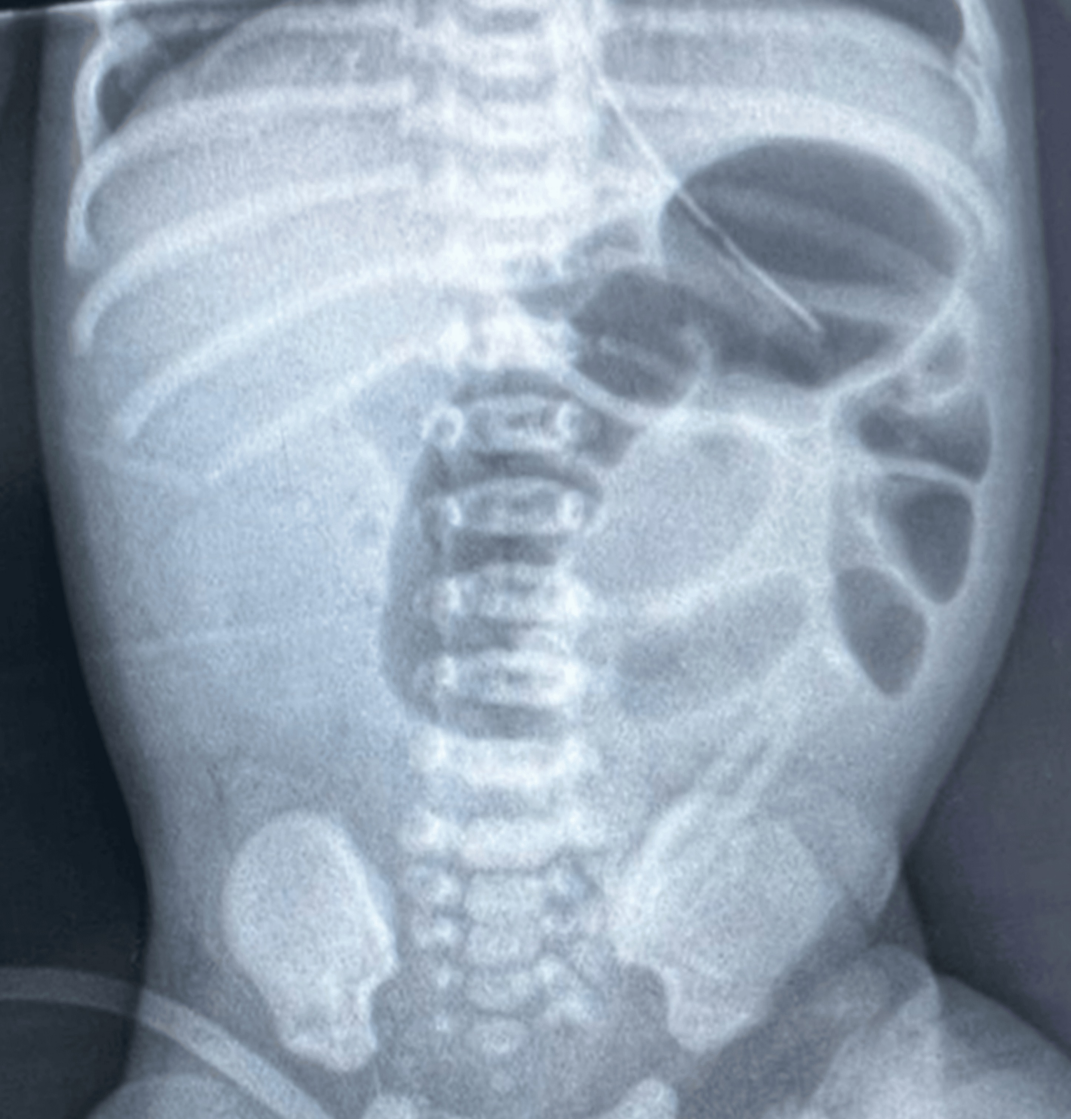
Plain abdominal radiography showing dilated small bowels.

**Figure 2:**
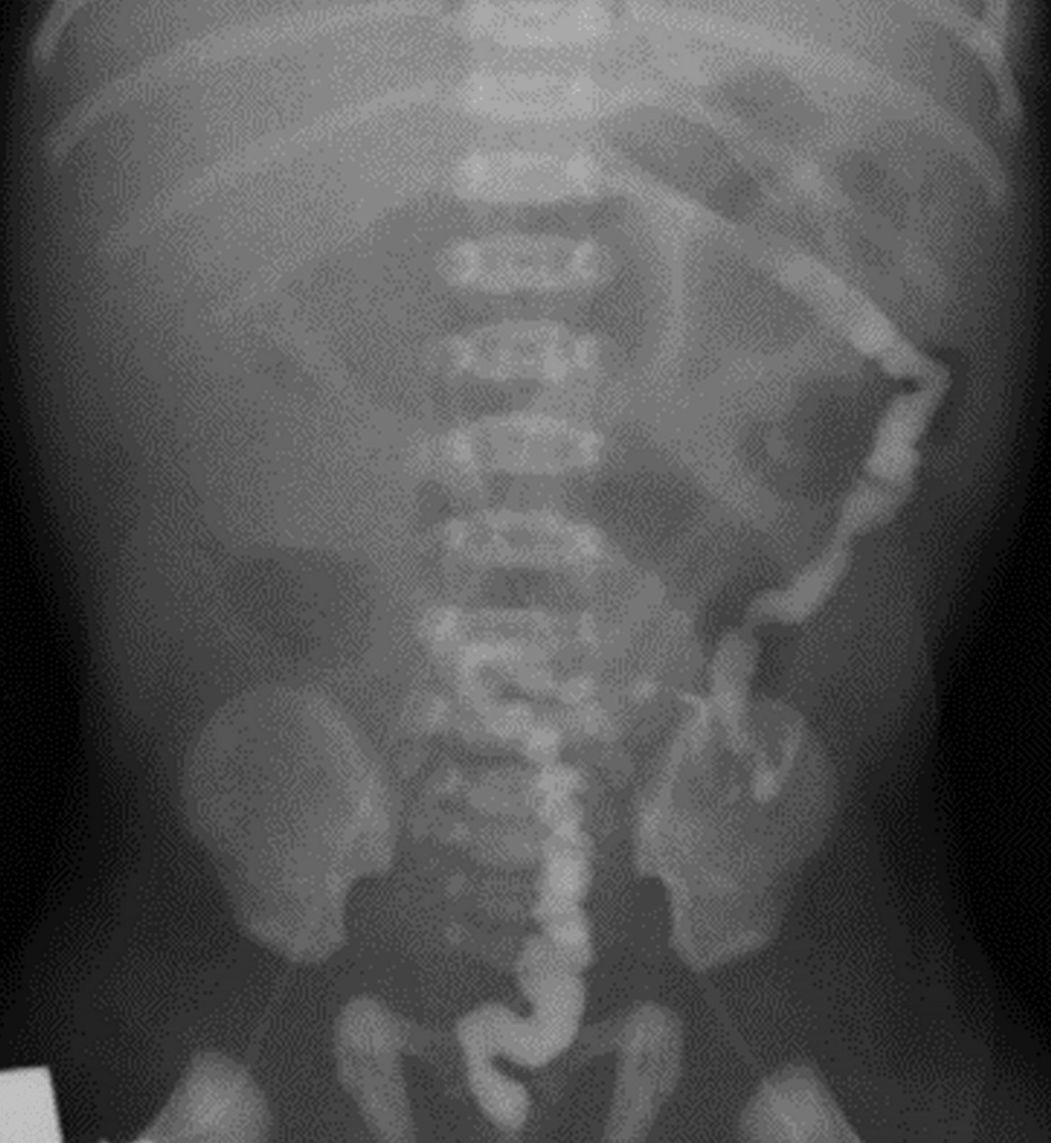
Lower gastrointestinal contrast study showing small calibre sigmoid, descending and part of transverse colon with abrupt tapering (features representing microcolon with colonic atresia).

A girl who was born at 40 weeks of gestation weighing 3800 g via spontaneous vaginal delivery with an Apgar score of 10 at one and five minutes after birth presented with persistent bilious vomiting on the first day of life. In addition to feeding intolerance, the abdomen was also markedly distended. Abdominal radiography showed dilated small bowels (Figure 1) and a gastrointestinal contrast study demonstrated microcolon, which indicated intestinal atresia (Figure 2).

**Figure 3:**
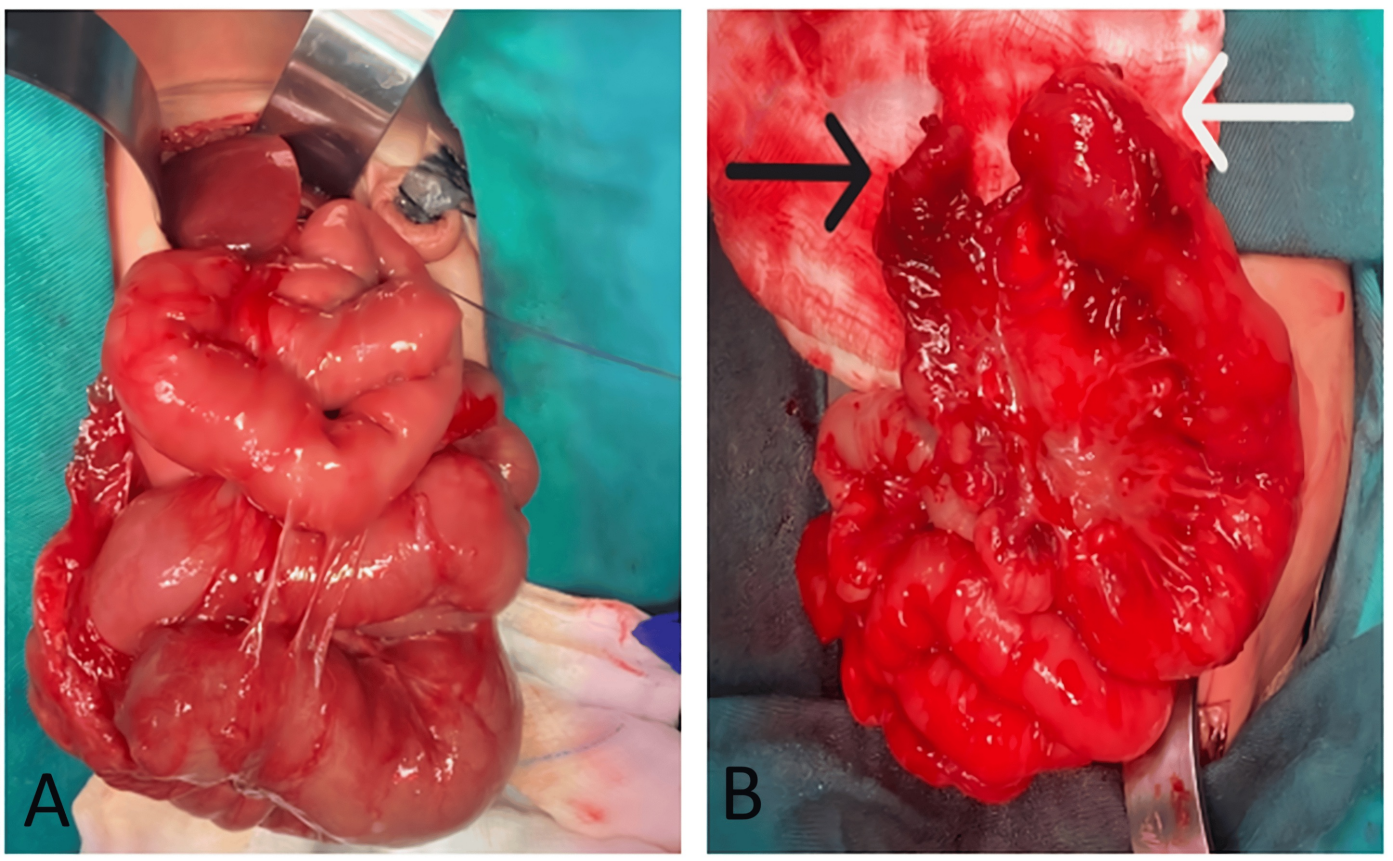
Type 3a jejunal atresia (with mesenteric gap). (A) Proximal dilated small bowels; (B) proximal small bowel atretic end (white arrow), distal small bowel atretic end (black arrow).

He was operated on day 3 of life. Intra-operatively type 3a jejunal atresia was found 50 cm from the duodeno-jejuno flexure with 5 cm of atretic segment (Figure 3).

**Figure 4:**
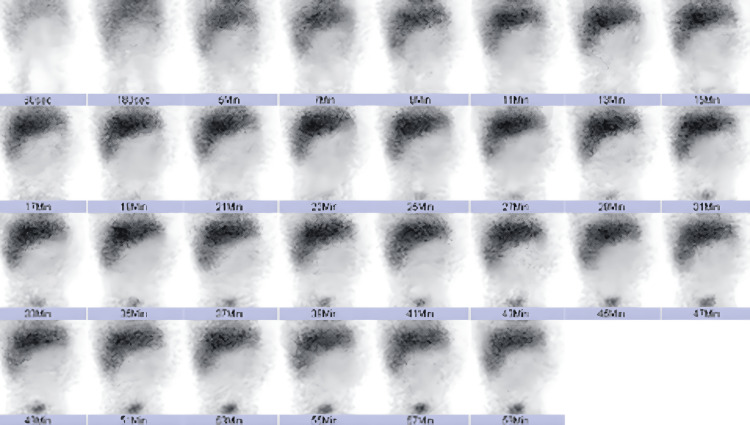
HIDA scan indicating no significant uptake of tracer activity in the extrahepatic biliary duct system, gallbladder and small bowel (good and homogenous uptake seen in the liver). HIDA: Hepatobiliary iminodiacetic acid.

Liver and gallbladder were grossly normal. Anastomosis was performed. Post-operatively, total parenteral nutrition was commenced and subsequently overlapped with enteral feeding. She tolerated feeding but noted increasing jaundice with clay-coloured stools on day 14 of life (post-operative day 9). The ultrasound abdomen performed could not visualize the gallbladder or common bile duct. Magnetic resonance cholangiopancreatography done on day 28 of life was inconclusive. A hepatobiliary iminodiacetic acid (HIDA) scan done on day 45 of life showed biliary obstruction with no tracer activity in the gastrointestinal tract at four and 24 hours post-injection which was suggestive of BA (Figure 4).

On day 56 of life, she was planned for Kasai hepatico-porto-enterostomy. Intra-operative findings revealed a type 3 BA. Only a thin fibrous cord of tissue was found in the remainder of the extra-hepatic biliary system. Therefore, on-table cholangiography was not performed. A 45 cm Roux-en-Y porto-enterostomy was created in a retrocolic manner. Post-operatively, she recovered uneventfully with an improvement of clinical jaundice, tolerated enteral feeding, and passing of brownish stool. She was discharged well but still jaundiced on post-operative day 9.

## Discussion

JIA remains one of the common causes of intestinal obstruction in neonates. It is due to vascular disruption causing ischaemic necrosis of a segment of the intestine. Subsequently, resorption of the necrotic segment in-utero would leave behind a blind proximal and distal end of the intestine with a mesenteric defect in between. Causes of in-utero vascular disruptions include intussusception, intestinal herniation, volvulus, omphalocele and gastroschisis. It is seen in 1 in 5000 to 1 in 14000 live births [1,4,5]. JIA is usually not associated with any other organ anomalies unless directly involved with the intestine. Otherwise, JIA occurs independently [4]. Where BA is concerned, its association with intestinal atresia is rate and the most frequent type of intestinal atresia associated with BA is duodenal atresia [6].

JIA typically does not exhibit aberrations in organ systems other than those specifically related to the gastrointestinal tract. There have been recorded instances of intestinal atresia in conjunction with cardiovascular anomalies, although it is typically observed as a separate abnormality unless it is accompanied by duodenal atresia or hindgut abnormalities.

The potential correlation between BA and duodenal deformity can be inferred based on their shared characteristics in embryonic development. In certain instances, such as the current scenario, it has been observed that the gallbladder is situated in its typical anatomical location and exhibits normal dimensions during the initial laparotomy. Additionally, the presence of bile content has been clearly identified during nasogastric suction. However, it is worth noting that acholic stool persists and total bilirubin levels remain elevated following the surgical procedure. In the current case, there was suspicion of a postnatal infection caused by bacteria or viruses, characterised by the presence of fever and eruption. It is postulated that the association between BA and JIA may have an aetiology originating during the perinatal or postnatal period, and may be linked to either a viral or bacterial etiological factor.

The survival rate at six months for patients with BA who have undergone a prior portoenterostomy procedure is reported to be between 75 to 82% [5,6]. Additionally, the survival rate for all patients at three years is documented to be 75% [5,6]. The prevailing consensus suggests that cystic type I BA is associated with a favourable prognosis. The age at which a patient undergoes BA surgery is a significant determinant of the outcome. The survival rate for BA in the United States between 1976 and 1989 exhibited varying percentages based on the age at which children received treatment; children treated within the first 30 days had a survival rate of 62.5%, while those treated between 31 and 60 days had a lower survival rate of 43.6% [1,4]. Infants treated at a later stage, specifically beyond 90 days of age, had the lowest survival rate of 28.6% [2,4,5]. In contrast, within the cohort of BA patients diagnosed with JIA in Japan, it was observed that none out of one individual who received treatment during the first 30 days of diagnosis lived. However, two out of five patients who were treated between 31 and 60 days after diagnosis were found to have survived. Furthermore, three out of six patients who received treatment beyond 61 days following diagnosis were observed to have survived. Consequently, the age at which BA surgery was performed did not demonstrate predictive significance in Japan [2,5]. The occurrence of recurrent cholangitis has a negative impact on the outcomes of surgical procedures.

Another area of concern pertains to the care of short bowel syndrome in BA. This condition necessitates the administration of prolonged total parenteral nutrition and has been demonstrated to elevate the likelihood of cholestasis, which can ultimately result in liver cirrhosis, sepsis, and an augmented risk of mortality. Preventing the occurrence of ascending cholangitis poses significant challenges in individuals with BA and JIA as a result of the presence of a truncated and diminutive intestinal tract. Hence, the survival rate among individuals with BA and JIA is much lower in comparison to the survival rate observed among all BA patients [7,8]. 

## Conclusions

Intestinal atresia is not usually associated with other organ system anomalies and usually occurs as a single independent anomaly. In cases where intestinal atresia occurs in combination with biliary atresia, duodenal atresia is found to be the most prevalent type of intestinal atresia. On the other hand, the incidence of jejuno-ileal atresia in cases of biliary atresia is reported to be extremely rate. Despite the identification of several potential reasons, including developmental deformity, perinatal viraemia, toxicity of bile components, and architectural anomalies in the hepatobiliary duct system, the precise aetiology of the condition remains elusive.
